# Spatial patterns and associations of tree species at different developmental stages in a montane secondary temperate forest of northeastern China

**DOI:** 10.7717/peerj.11517

**Published:** 2021-06-03

**Authors:** Jia Liu, Xuejiao Bai, You Yin, Wenguang Wang, Zhiqiang Li, Pengyu Ma

**Affiliations:** 1 College of Forestry, Shenyang Agriculture University, Shenyang, China; 2 Research Station of Liaohe-River Plain Forest Ecosystem, Chinese Forest Ecosystem Research Network (CFERN), Shenyang Agricultural University, Tieling, China; 3 Qingyuan Forest CERN, Chinese Academy of Sciences, Shenyang, China

**Keywords:** Spatial pattern, Spatial association, Developmental stages, Secondary forests, Point pattern analysis, Aggregation, Habitat heterogeneity, Competition, Plant size, Species coexistence

## Abstract

**Background:**

Secondary forests have become the major forest type worldwide. Research on spatial patterns and associations of tree species at different developmental stages may be informative in understanding the structure and dynamic processes of secondary forests.

**Methods:**

In this study, we used point pattern analysis to analyze the spatial patterns and associations of tree species at seedling, sapling and adult stages in a 4ha plot in the montane secondary temperate forest of northeastern China.

**Results:**

We found that species showed similar patterns at seedling, sapling and adult stages, and aggregation was the dominant pattern. The spatial patterns of tree species were mainly affected by habitat heterogeneity. In addition, the strength of positive or negative associated pattern among tree species would decrease with developmental stages, which attributed to neighborhood competition and plant size increasing.

**Conclusions:**

Our results indicated that the spatial patterns and associations of tree species at seedling and sapling stages partly reflected that at adult stage; habitat heterogeneity and neighborhood competition jointly contributed to species coexistence in this secondary forest.

## Introduction

A general description of spatial patterns of woody plants is a necessary step to understand structure and dynamics of forest community (Akhavan et al., 2012; Perry et al., 2002; Watt, 1947; Zhang et al., 2010a). Increasing studies confirmed that most species were not randomly distributed; they either aggregated or dispersed (Condit et al., 2000; Watt, 1947; Wiegand, Gunatilleke & Gunatilleke, 2007). This observation has attracted a large number of scholars to investigate the pertinence of the spatial patterns regarding species coexistence and the maintenance of biodiversity (Hurtt, 1995; Murrell, Purves & Law, 2001; Szmyt & Tarasiuk, 2018). When species were aggregated distributed, the frequency of interspecific encounters decreased, promoting species coexistence (Stoll & Prati, 2001). Therefore, the spatial pattern of species has always been one of the important contents of ecological research (McIntire & Fajardo, 2009; Wiegand & Moloney, 2004). In recent years, many studies in forest ecosystem have been related to spatial patterns (Fajardo, Goodburn & Graham, 2006; Guo et al., 2014; Law et al., 2009), and found that the aggregation distribution often occupied a large proportion in tropical, subtropical and temperate forests (Guo et al., 2013; Nguyen et al., 2016; Wang et al., 2010; Zhang et al., 2013b). Many factors could affect the aggregated pattern, such as habitat heterogeneity, seed dispersal limitation, intraspecific competition or combinations of these factors (Inman-Narahari et al., 2014; Lara-Romero et al., 2016; Zhang et al., 2010b).

Spatial pattern can aﬀect species spatial associations (Callaway & Walker, 1997; Zhang et al., 2010b), which refers to the interrelation of spatial distributions of different populations (Zhang et al., 2013a). The negative interactions among species would reduce the density of heterospecific neighbours at short distances, while positive interactions would present an opposite effect (Martinez et al., 2010). Research on species associations plays an important role in understanding the interactions and ecological relationships between species (Wang et al., 2010), and also provides information on dynamics of the component species (Wang et al., 2016). In tropical, subtropical and temperate forests, many studies found positive associations among tree species (Lan et al., 2012; Ledo, 2015; Luo et al., 2012; Martinez et al., 2010). Actually, the spatial association among species was the result of species interaction and species adaptation to the environment (Wang et al., 2010).

The spatial patterns and associations of tree species would change with different growth stages of tree species (Gu et al., 2019; Ledo, 2015; Zhou et al., 2019). Studies found that most species tended to be aggregated at seedling stage, while they tended to be regular or random at adult stage (Condit et al., 2000; Getzin et al., 2006; Stoll & Bergius, 2005). The aggregation of seedlings was mainly due to seed dispersal and non-regular germination (Akhavan et al., 2012), while the spatial distribution of adults was mainly affected by species competition and habitat heterogeneity (Li et al., 2009). In temperate forests, the interactions of different life strategy species changed from positive association at juvenile stage to negative association at adult stage (Liu, Li & Jin, 2014), and the percentage of negative associated species pairs increased with development stages (juvenile, medium and large tree) (Chai et al., 2016), which were affected by habitat heterogeneity, plant size and species interaction (Getzin et al., 2008; Wang et al., 2010; You et al., 2010). In general, the spatial patterns and associations of woody plants were influenced by the combined effects of biological characteristics and environmental factors. Hence, analyzing spatial patterns and associations plays a vital role in understanding species interaction and ecological processes (Hou et al., 2004; Lan et al., 2012; Li et al., 2014; Wiegand, Gunatilleke & Gunatilleke, 2007), especially in different growth stages.

Secondary forests, regenerated from primary forests after extreme natural or anthropogenic disturbances (Zhu & Liu, 2007), were significantly different in species composition and stand environment compared with primary forests (Zhu, 2002). Currently, secondary forests have become a major forest type in many regions worldwide (Brown & Lugo, 1990; Finegan, 1996); they account for 70% of the natural forests in northeastern China (Yang et al., 2010). Research on spatial patterns and associations of tree species at different developmental stages may be informative in understanding the structure and dynamic processes of secondary forests (Gu et al., 2019). In this study, we analyzed spatial patterns and associations of tree species at different developmental stages (seedling, sapling and adult) in a montane secondary forest of eastern Liaoning Province, China. The following questions were addressed: (1) How do the spatial patterns and associations of tree species change with different developmental stages in this secondary forest? (2) Which ecological processes (e.g., seed dispersal limitation, habitat heterogeneity, facilitation and competition between species) could structure these patterns and associations?

## Materials & Methods

### Study area and data collection

The study area is in Benxi Manchu autonomous county, located at Liaoning province, in northeastern China (N41°05′42.10″,E124°30′38.66″). This area is characterized as temperate monsoonal climate. Total annual precipitation is 700–1,000 mm, and mean annual temperature is 6–8 °C. The frost-free period is 130 days. Main tree species include *Quercus mongolica*, *Acer mono*, *Tilia amurensis*, *Fraxinus mandshurica* and *Juglans mandshurica*.

The 4 ha (200 m × 200 m) plot was established in 2017 to monitor long-term dynamics in a montane secondary forest of eastern Liaoning Province, China. The elevation of this plot ranges from 759.3 m to 900.8 m, with a mean elevation of 827.5 m. In this plot, all woody stems with ≥1 cm diameter at breast height (DBH, 1.3 m above the ground) were tagged, mapped, measured and identified in 2017, and all woody stems with <1 cm DBH were also tagged, mapped, measured and identified in 2018. According to the census in 2017, we recorded 14,036 individuals belonging to 24 families, 34 genera, and 46 species. The species with ≥20 individuals in each of three developmental stages (seedling, sapling and adults) were selected (Table 1). The individuals were classified into three developmental stages: seedling (DBH < 1 cm) for all species, sapling (1 cm ≤ DBH < 8 cm) and adult (DBH ≥ 8 cm) for large canopy tree species, such as *Fraxinus rhynchophylla* and *Tilia amurensis*, while sapling(1 cm ≤ DBH < 5 cm) and adult (DBH ≥ 5 cm) for small canopy tree species. There were 11 species at each developmental stage (Table 1).

**Table 1:** Number of individuals for analyzed species at seedling, sapling and adult stages.

Species	Number of seedlings	Number of saplings	Number of adults
*Acer mono*	1527	2614	555
*Ulmus laciniata*	633	1312	518
*Syringa reticulata*	1419	757	46
*Fraxinus rhynchophylla*	88	276	362
*Acer pseudo-sieboldianum*	266	405	207
*Padus racemosa*	718	220	30
*Carpinus cordata*	95	172	59
*Acer triflorum*	52	140	23
*Acer tegmentosum*	780	101	58
*Tilia amurensis*	98	93	64
*Sorbus alnifolia*	25	56	51

### Data analysis

Ripley’s K function K(r) is an important spatial pattern statistic, which is a cumulative distribution function within the distance of r (Getis & Franklin, 1987; Greig-Smith, 1952; Ripley, 1977). In this study, we used the pair correlation function *g(r)* to analyze the spatial patterns of tree species at different stages (Ripley, 1976). The function *g(r)* is a counterpart to the function *K(r)*:

$$K(r)=n^{2}\left|A\right|\sum_{i\neq j}\sum e_{ij}^{-1}(u_{ij})$$

$$g\left(r\right)=\frac{1}{2\pi r}\frac{dK\left(r\right)}{d\left(r\right)}$$

where r is the distance (ring in g(r)) rad, A is the area of study plot, uij is the distance between the focal tree (i) and its neighboring tree(j), n is the total number of points in the point pattern, uij = 1, if uij <rand 0 otherwise, and eij is the weighting factor for eliminating edge effect correction.

In this study, we used two models. Complete spatial randomness (CSR) assumes no interactions between objects (Wiegand & Moloney, 2004). Heterogeneous Poisson process (HPP) is aimed to eliminate the effect of habitat heterogeneity. In this null model, relationships between habitat heterogeneity and tree species are used to via a spatially heterogeneous intensity function, *λ(s)*. And the function varied with location *s*. The parametric model is fit as:

$$\lambda \left(s\right)=exp\left(\beta^{T}X\left(s\right)\right)$$

*X(s)* is a vector of environmental variables and *β* is a vector of regression parameters. The four topographic variables used in this study were slope, aspect, convexity and elevation.

For the univariate function g(r), the spatial scale was 0–50 m, and we used a ring width of one meter and used 199 Monte Carlo simulations of CSR and HPP to acquire pointwise critical envelopes, and the significance level was 0.01 (namely *p* ≤ 0.01). If the value of g(r) was above (or below) the upper limit of the confidence envelope, the spatial pattern indicates the aggregated (or regular) pattern at a given distance r, and within the confidence intervals indicated random pattern.

To investigate the spatial associations of tree species, we used bivariate pair correlation function g12(r), which is the extended g(r) function to multitype point patterns. g12(r) can be defined as the expected number of trees of species2 at spatial scale r of an arbitrary tree of species 1, divided by the intensity of species2 (Stoyan & Penttinen, 2000).

$$g_{12}(r)=\frac{1}{2\pi r}\frac{A}{n_{1}n_{2}}\sum_{i=1}^{n_{1}}\sum_{j=1}^{n_{2}}u_{ij}(e_{ij})$$

where uij is the distance between the focal tree of pattern 1, and its neighboring tree of pattern 2, n1 and n2 are the total numbers of trees in the patterns 1 and 2, respectively (Wiegand & Moloney, 2004). For g12(r), we also used a ring width of one meter and used 199Monte Carlo simulations of CSR and HPP to acquire pointwise critical envelopes and the significance level was 0.01 (namely *p* ≤ 0.01). If the value of g12(r) was above the upper (or below the lower) confidence limit, the relationship indicates that species are positively (or negatively) associated at the distance r, and within the confidence intervals indicated no interaction (Thioulouse et al., 1997; Wiegand, Jeltsch & Ward, 2000).

We used the package “spatstat” in R3.6.3 to conduct all spatial analyses.

## Results

### Intraspecific spatial patterns

**Figure 1:**
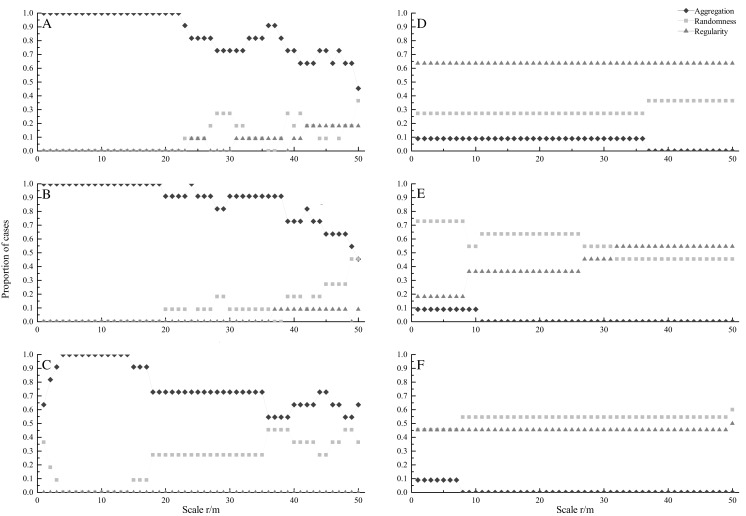
Proportion of species at seedling (A, D), sapling (B, E) and adult (C, F) stages showing significant aggregation (diamonds), random (squares), and regular (triangles) across different scales under CSR null model (A, B, C) and HPP null model (D, E, F).

At seedling stage, most species showed significant aggregated distributions under CSR (Fig. 1A). All species were aggregated at scales from 0 to 22 m. The percentage of aggregated species decreased with increasing spatial scales, and the minimum percentage was 45.5% at 50 m. On the contrary, the percentage of random and regular species increased at larger scales. Under HPP, regular distribution was the dominant pattern, and the percentage was 63.6% at scales from 0 to 50 m (Fig. 1D).

At sapling stage, all species were aggregated at scales from 0 to 19 m (Fig. 1B). The percentage of aggregated species decreased with increasing scales, and the minimum percentage was 45.5% at 50 m, whereas the percentage of random distributed species increased with increasing scales. Under HPP, random distribution was the dominant pattern. The percentage of random distributed species was 72.7% at small scales (0–8 m) and decreased with increasing scales. While, the percentage of regular distributed species increased with increasing scales, and the maximum percentage was 54.5% (Fig. 1E).

At adult stage, most species also showed aggregated distribution under CSR (Fig. 1C). Similar to seedling and sapling stage, the percentage of aggregated distributed species decreased with increasing scales, while the percentage of random distributed species increased with increasing scales. Under HPP, random and regular distributions were dominant patterns (Fig. 1F), the average percentages were 53.4% and 45.5% respectively.

### Interspecific associations

**Figure 2:**
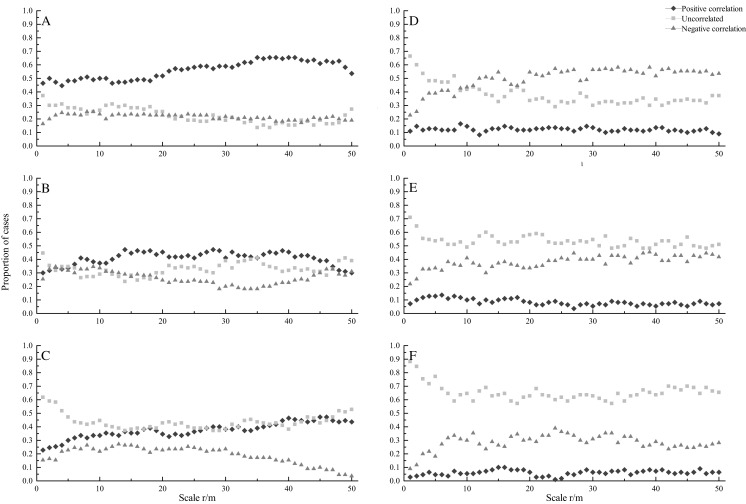
Proportion of species at seedling (A, D), sapling (B, E) and adult (C, F) stages showing significant positive correlation (diamonds), uncorrelated (squares), and negative correlation (triangles) across different scales under CSR null model (A, B, C) and HPP.

A total of 110 pairs of species were investigated in this study. At seedling stage, most species showed positively correlated under CSR. The percentage of positively correlated species pairs increased with increasing spatial scales, ranging from 44.5% to 65.5%, while the percentage of uncorrelated and negatively correlated species pairs decreased with increasing scales (Fig. 2A). Under HPP, most species showed uncorrelated at scales from 0 to 8 m, while negatively correlated at scale from 12 to 50 m. The percentage of negatively correlated species pairs increased with increasing scales, ranging from 22.7% to 58.2%, while the percentage of uncorrelated species pairs decreased with increasing scales, ranging from 66.4% to 29.1%. The percentage of positively correlated species pairs showed a relatively flat trend (Fig. 2D).

At sapling stage, under CSR, the percentages of uncorrelated, negatively and positively correlated species pairs were almost the same at scales from 0 to 6 m, while the percentages of uncorrelated and positively correlated species pairs were higher than that of negatively correlated species pairs at larger scales (Fig. 2B). Under HPP, the percentage of uncorrelated species pairs was higher. The percentage of uncorrelated species pairs decreased with increasing scales, ranging from 70.9% to 48.2%, while the percentage of negatively correlated species pairs increased with increasing scales, ranging from 21.8% to 45.5% (Fig. 2E).

At adult stage, under CSR, the percentage of uncorrelated species pairs was higher. The percentage of uncorrelated species pairs decreased with increasing scales, ranging from 61.8% to 37.3%, while the percentage of positively correlated species pairs increased with increasing scales, ranging from 22.7% to 47.3%. The percentage of negatively correlated species pairs also showed a decreasing trend, ranging from 15.5% to 3.6% (Fig. 2C). Under HPP, the percentage of uncorrelated species pairs was much higher than that of negatively and positively correlated species pairs, the average percentages were 65.4%, 28.5% and 6.1%, respectively (Fig. 2F).

## Discussion

### Intraspecific spatial patterns

In secondary forests, seed dispersal limitation and habitat heterogeneity were found to be the most important factors determining the distribution patterns of species (Collet, Manso & Barbeito, 2017; Shen et al., 2009; Wang et al., 2016). Actually, the spatial pattern was affected by seed dispersal of tree species at small scales, while habitat heterogeneity is the main factor affecting the spatial pattern at larger scales (Yuan et al., 2011). Our study found that under CSR most species showed aggregated pattern at seedling, sapling and adult stages. The percentage of aggregated distributed species at seedling, sapling and adult stages were 100%, 100% and 93.6% at scales from 0 to 10 m, and still up to 65.3%, 66.1% and 63.6% at scales from 40 to 50 m under CSR. After eliminating the effect of habitat heterogeneity, the regular or random distribution at the three stages became the dominant pattern under HPP. Therefore, habitat heterogeneity played an important role in the spatial patterns of tree species. Tree species preferred a certain habitat to form the aggregated pattern (Luo et al., 2012). Previous studies found that habitat heterogeneity caused different topography, soil nutrients and light intensity, thus affected the spatial patterns of tree species (Getzin et al., 2008; Song, Li & Zhang, 2010; Yuan et al., 2011). For example, Fang et al. (2017) found that topographic and soil played important roles in the spatial patterns of tree species in evergreen broad-leaved forests. Because habitat heterogeneity can increase plant density in locally suitable environments to promote species aggregation, the uneven distribution of limited resources may affect species patterns (Getzin et al., 2008).

Condit et al. (2000) found that tree species tended to be regularly or randomly distributed with the growing process of woody plants. There is no enough evidence to show that the aggregated pattern at seedling stage would persist up to adult stage (Aparajita & Rawat, 2008; Seidler & Plotkin, 2006). But many studies have shown that most species were aggregated at adult stage (Li et al., 2009; Nguyen et al., 2016; Wang et al., 2010). Our study found that species had certain similarity in spatial patterns at seedling, sapling and adult stages, and also found some differences in specific scale. Under CSR, most species were aggregated at seedling, sapling and adult stages, which were consistent with other temperate forest (Liu, Li & Jin, 2014). The average percentages of aggregated distributed species were85.6%, 88.4% and 76.7% at seedling, sapling and adult stage, which partly supported the previous studies that found the percentage of aggregated distributed species would decrease with increasing life stages (Lan et al., 2012; Lan et al., 2009; Zhu et al., 2013). Density-dependent mortality of the offspring can contribute to this trend in the intraspecific spatial pattern.

### Interspecific associations

The aggregated, random and regular patterns demonstrated that the ecological relationships between two species were mutually beneficial, not obvious, and mutually exclusive, respectively (Phillips & MacMahon, 1981). Spatial patterns would play a key role in the interactions between species, and these interactions affect ecological processes related to species dynamics, such as growth, regeneration, and death (Bieng et al., 2013). In our study, the percentage of species pairs with positive correlation would decrease with life stages under CSR. After eliminating the effect of habitat heterogeneity, under HPP, the negative correlated and uncorrelated patterns became the dominant patterns at the three stages. The results indicated that habitat heterogeneity was one of the important factors affecting the spatial associations among tree species. Under HPP, the negative correlated pattern was the dominant pattern at the seedling stage, while the uncorrelated pattern was the dominant pattern at the sapling and adult stages. The reason may be that species at seedling stage were more sensitive to habitat heterogeneity and neighborhood plants than species at adult stage (Suding & Goldberg, 1999). Neighborhood interactions for resource competition could affect ecological niche differentiation. Species may compete for resource when they need the same habitat conditions (Parrish & Bazzaz, 1982). While, the competition between species is more intense at seedling stage, which may due to limited distance between individuals at seedling stage (Collet, Manso & Barbeito, 2017). Therefore, the strength of spatial association would decrease with the growing process (Gu, Gong & Li, 2017).

The differences of interspecific association at different growth stages may be affected by the size of plants (Shen et al., 2016). The greater differences of plant size among individuals, the weaker positive correlation between individuals were existed (You et al., 2010). In our study, under CSR, the percentage of species pairs at seedling stage with positive correlation (56.0%) was higher than that at sapling and adult stage (40.8% and 37.5%). The positive correlation among species at seedling stage was more intense than that at sapling and adult stages, because the individual size difference of seedlings was not obvious than that at sapling and adult stages. In addition, a larger individual may be more competitive to soil nutrients, light, and other resources than a smaller individual. When individuals reached to adult stage, the positive correlation may disappear because of competition (Martinez et al., 2010). Moreover, we also found that the dominant pattern changed from negative correlated pattern at seedling stage to uncorrelated pattern at sapling and adult stages under HPP, partly because a large number death of individuals at seedling stage lead to the distance between individuals increased and eliminate negative correlation at sapling and adult stages.

## Conclusion

We found that species showed similar spatial patterns at seedling, sapling and adult stages. Although the degree of aggregation decreased with developmental stages, aggregation was the dominant pattern, which mainly affected by habitat heterogeneity. The strength of interspecific positive or negative associated pattern would decrease with the developmental stages, which attributed to interspecific competition and plant size increasing. Our results indicated that habitat heterogeneity and neighborhood interactions jointly contributed to species coexistence in this secondary forest. Although understanding spatial patterns and associations of tree species would reveal mechanisms of interspecific replacement in the process of forest development and provide a theoretical basis for vegetation restoration and reestablishment, the interspecific replacement mechanisms, species coexistence and relevant ecological processes need to be observed for a long time. We believe that the results of this study would provide information on spatial patterns and associations of tree species in secondary forests, which can be used to implement the protection and recovery of secondary forests.

## Supplemental Information

10.7717/peerj.11517/supp-1 Supplemental Information 1 The original measurement data at adult stage.

10.7717/peerj.11517/supp-2 Supplemental Information 2 Sapling.

10.7717/peerj.11517/supp-3 Supplemental Information 3 The original measurement data at seedling stage.

## Additional Information and Declarations

### Data Availability

The following information was supplied regarding data availability: The raw measurements are available in the Supplemental Files.
